# Incorporating Data Sets With Multiple Sources of Uncertainty in Integrated Species Distribution Models

**DOI:** 10.1002/ece3.73185

**Published:** 2026-04-09

**Authors:** Fiona Lunt, C. Lane Scher, Riley O. Mummah, David A. W. Miller

**Affiliations:** ^1^ Department of Ecosystem Science and Management The Pennsylvania State University University Park Pennsylvania USA; ^2^ Eastern Ecological Science Center U.S. Geological Survey Turners Falls Massachusetts USA

**Keywords:** community science data, data filtering, data integration, false positive models, integrated species distribution models

## Abstract

Data integration methods aim to improve species distribution estimates by incorporating multiple sources of uncertainty across datasets. Two major sources of uncertainty are: (1) variation in sampling effort across space and within datasets, and (2) variation in reliability associated with data collection protocols or timing among datasets. Our goal was to evaluate how different approaches to address these uncertainties influence predictive performance of integrated models. We modeled distributions of four bird species using three datasets that differed in sampling design. We examined three strategies to reduce uncertainty: (1) filtering data, (2) incorporating functions that account for uncertainty in observation models, and (3) varying how datasets are integrated into a single estimate. We first examine methods to account for variable effort in observations, focusing on both spatial differences in sampling intensity and effort given to a single observation record. We then examine approaches to account for data sets with differing reliability. Sampling effort was best addressed through conservative filtering, including spatial thinning and excluding observations with highly variable effort. Next, we considered how to account for potential false positive detections—due to either misidentification or changes in distributions. We found that treating less reliable data as a covariate, an approach previously suggested for data integration that can greatly speed up model fitting, performed well. Other effective approaches included directly modeling false positive rates and complete exclusion of less reliable data sets. Our results provide insights into best practices in integrated modeling for handling uncertainty in integrated models. We demonstrate the flexible options available when using integrated models to address uncertainty.

## Graphical abstract

We explore best practices for building integrated species distribution models—using data collected for forest birds as a test case. We demonstrate that a combination of data filtering methods to reduce less reliable data and model‐based adjustments to account for data set specific differences improves predictions from integrated models.

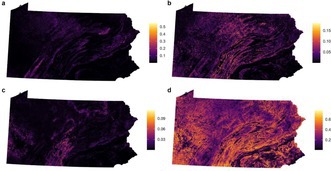

## 1 Introduction

Accounting for reliability and completeness of species observations data is essential for building more accurate species distribution models (SDMs) that can better support conservation applications and ecological inquiry (Guillera‐Arroita et al. 2015). Recent advances in methods and algorithms used for fitting models, coupled with rapidly increasing data availability, have greatly enhanced the flexibility and utility of distribution models (Miller et al. 2019). However, some data sources, such as participatory science efforts and historic museum collections, pose challenges for reliable inference due to how they are collected. Unstructured protocols in participatory data collection lead to uneven sampling effort across space, whereas variation in behavior and expertise of observers can produce false positives due to misidentification (Johnston et al. 2021). Despite these limitations, participatory science projects often collect data across a much greater spatiotemporal extent than structured survey efforts, providing invaluable information about species' ranges (Johnston et al. 2021; Zulian et al. 2021; Binley et al. 2024). Similarly, historic data such as museum records can fill in gaps in needed to estimate distribution extents but may no longer accurately reflect current distributions (Davis et al. 2023).

As data sources with distinct characteristics are combined, statistical methods that account for data set‐specific challenges are crucial for accurate inference (Fletcher et al. 2016; Zulian et al. 2021). Data integration methods have been proposed as a framework for building species distribution models where multiple data sets with different observation methods are combined (Dorazio 2014; Miller et al. 2019; Isaac et al. 2019). These methods account for the observation process used to generate each unique data set, in contrast with methods that simply pool multiple data sets with the assumption that all data sets can be described by the same statistical model (Fletcher et al. 2019). Previous studies have demonstrated how data integration can improve a model's predictive ability, produce more reliable estimates of abundance and distribution, and improve inference for rare species (Schaub and Abadi 2011; Fithian et al. 2015; Fletcher et al. 2016, 2019; Giraud et al. 2016; Pacifici et al. 2017; Koshkina et al. 2017). However, developing best practices for integration methods requires thorough testing of different methodological approaches, including how to account for heterogeneous effort and misidentification, and determining which statistical approach should be used to combine data streams to generate a single estimate of the species' distribution.

Data are often collected from systematic, structured surveys or unstructured, opportunistic observations. Structured surveys typically use protocols that standardize survey effort, generally at the expense of reduced spatial coverage (McClintock et al. 2010; Miller et al. 2012; Robinson et al. 2020). In contrast, in non‐systematic survey protocols, such as with many participatory data sources and museum data collections, effort is likely to be highly variable among observations and clustered in specific geographic regions. The participatory science platform eBird (Sullivan et al. 2009), for example, is among our best global biodiversity observation data sets but includes widely varying survey effort among samples, significant spatial clustering of observations, and variable observer expertise (Strimas‐Mackey et al. 2020). Integration with structured surveys can strengthen the inference derived from unstructured data sources (Fithian et al. 2015; Miller et al. 2019).

Methods to account for heterogeneity in effort generally fall into two categories: filtering data to reduce variation or accounting for variation in effort as part of the statistical model. Filtering addresses variable effort by reducing the variation among observations across space before the observations enter the model (Pacifici et al. 2019; Fink et al. 2020; Robinson et al. 2020). Reducing variation in detection, which is generally correlated with effort, by restricting observations to a limited range of survey distance, survey duration, and number of observers can improve the reliability of distribution estimates (Strimas‐Mackey et al. 2020). Finally, spatial filtering may improve inference by reducing overfitting that results from clusters of observations in a small geographic region (Steen et al. 2020; Strimas‐Mackey et al. 2020). While these filters can increase the consistency within participatory science data, they also reduce the amount of data available, and their ad hoc nature may make them less appealing than model‐based approaches.

An alternative strategy to control for variation in effort is to link effort to the probability of detecting a given species within the statistical model. When effort varies greatly among sample units, detection can be approximated using an observation model that directly estimates effort and its influence on detection (Stauffer et al. 2018; Miller et al. 2019; Zulian et al. 2021). Detection rates should increase with survey effort, so survey duration and distance traveled are often used as proxies for effort (Stauffer et al. 2018; Miller et al. 2019; Zulian et al. 2021). Other factors like time of day and day of year, which are related to bird activity, may also influence the probability of detection and can be included in the observation model (Skirvin 1981; Diefenbach et al. 2007).

After examining approaches to account for effort, we consider strategies for integrating multiple data streams when one data set is less reliable than another. The reliability of a data set may decrease if it includes species misidentifications or if changes in a species distribution have occurred since the data were collected. Pacifici et al. (2017) suggests two approaches to address reliability. The first is to include a false positive detection rate for a less reliable data set within a standard joint‐likelihood model (Miller et al. 2013; Pacifici et al. 2017). False positives may be more likely to occur in participatory science data sets, but they can also arise in historic data sets if the records are not representative of a species' current distribution. The second approach they suggest is to summarize the data from the less reliable data set to be used as a covariate in the species distribution model. This strategy allows the less reliable data set to inform predictions indirectly, based on how well observations correlate with observations from the more reliable data sets. Comparisons of these methods will help elucidate when each strategy works best.

We examine how different methods for combining data sets with heterogeneous effort and less reliability with structured data affect the fit and predictive ability of integrated distribution models. We focus on two questions. First, what is the optimal way to account for variable effort in an integrated SDM? We test a series of filtering criteria intended to reduce variation in effort as well as several methods of directly accounting for effort within the model. Second, what is the optimal way to integrate less reliable and trusted data sets in a model? We compare full integration of the less reliable data set, treating the less reliable data set as a covariate, and excluding the less reliable data set entirely. As our case study, we construct distribution models for four bird species of conservation need in the state of Pennsylvania, USA (PGC‐PFBC 2015), and determine which approaches improve predictive ability of our models.

## 2 Materials and Methods

### 2.1 Data

We analyzed data for four bird species, Canada Warbler ( *Cardellina canadensis* ), Cerulean Warbler ( *Setophaga cerulea* ), Golden‐winged Warbler ( *Vermivora chrysoptera* ), and Wood Thrush ( *Hylocichla mustelina* ). All are listed on the Pennsylvania State Wildlife Action Plan as species of greatest conservation need (PGC‐PFBC 2015). The four species differ in relative abundance, with Golden‐winged Warbler being least common and Wood Thrush most abundant, as well as their general distribution and habitat associations, allowing us to compare the consistency of our results across a range of scenarios. Data used in our analyses were drawn from three data sets described in the following, and we limited observations to the state of Pennsylvania, USA, which allowed for similar spatial coverage of all three data sets. All datasets are conditional on where sampling occurs, so that 0's represent a site where no individuals were observed of the species (rather than sites where sampling did not occur). For our analyses, we treat all data as binary (the species was detected or not detected) rather than as counts. Counts greater than one were rare in our data set.

The North American Breeding Bird Survey (BBS) is a roadside survey that has been run annually since 1966 at pre‐selected routes that each consist of 50 point‐count stops located 800 m apart (Sauer et al. 2013). A 3‐min survey is conducted at each stop, and all birds heard or seen within a 400‐m radius are recorded. We used BBS data for Pennsylvania that included georeferencing for each individual stop, which allowed us to use observations at the site‐level rather than route‐level. Site‐levels summaries are only available back to 1997. We used only observations from routes that met the BBS quality criteria for inclusion (i.e., RunType = 1).

The Pennsylvania Breeding Bird Atlas (BBA) data set includes point‐count surveys, conducted at pre‐selected locations distributed evenly across the entire state and which were conducted from 2004 to 2009 during peak breeding season each year (late May to early July). For these surveys, observers recorded singing males of all species, denoting whether the observation was for a bird < or > 150‐m radius from the point count locations. Point counts lasted 6 min and 15 s and observations were divided into five 75‐s intervals (Wilson et al. 2012; Paton et al. 2019). We treated the five intervals as repeated surveys of the same location.

eBird is a participatory science program developed by the Cornell Lab of Ornithology that allows users to submit observations (“checklists”) of bird counts and detections (Sullivan et al. 2009). The sampling protocol is semi‐structured: individual records vary in duration, distance traveled, observation method (e.g., stationery or traveling), time of day, and number of observers, but users are encouraged to enter this information with their sightings. The recorded effort variables allow checklists to be vetted and filtered and can be used to estimate actual effort associated with the observations (Strimas‐Mackey et al. 2020). For all analyses, we only included complete checklists, and we treated observations as detection/non‐detection data rather than as counts. We limited data to lists collected starting in 2014 and only included observations during the peak breeding season (May 25th to July 5th), consistent with the survey window for the other two data sets. For our reference models (see *Reference model*), we followed the standard recommendations from Strimas‐Mackey et al. (2020): we filtered by effort to include only checklists < 5 h in duration, < 10 km in distance traveled, and < 10 observers. Unlike the other data sets, where surveys are designed to create uniform coverage across the spatial extent, eBird data can include extreme spatial clustering of samples. Our default filter to create greater spatial balancing of the data was to create a 1‐km hexagonal grid over the extent of Pennsylvania and selecting one checklist per grid cell, discarding the rest. As explained in the following, we compare these defaults to other filtering methods to determine what filtering decisions lead to the best predictive outcomes.

Environmental variables used as covariates in the models were elevation, slope (30‐m DEM, U.S. Geological Survey), and precipitation (800‐m resolution, 1981–2010 30‐year Normals, PRISM Climate Group) taken at the point of the observation. For landscape variables we included the proportion of developed land, proportion of forested land (30‐m resolution, National Land Cover Database), and total length of local and state roads (PennDOT) within a 400‐m buffer around each survey point. All environmental covariates were standardized by subtracting their respective means and dividing by their standard deviations. In addition, we considered spatial effects based on latitude, longitude, and their interaction to explicitly account for unexplained spatial autocorrelation (Hefley et al. 2017).

### 2.2 Reference Model

#### 2.2.1 Process Model

A complete list of the models we fit to our data can be found in Table 1, with detailed descriptions in the following section. We first describe our reference model which includes a process model to describe the species distribution and observation models that link the process model to each of our three data sources. The reference model was our starting point for comparison to other models. All model comparisons were developed to determine which decisions to account for sources of uncertainty led to the best predictive output. We chose to explore decisions that span the analytical process included what data were included when fitting models (e.g., how to filter eBird data and whether to include historic records), how the structure of the model was formulated (e.g., whether to account for effort in an observation model), and how the data from multiple data sets were integrated (e.g., using a joint‐likelihood versus a covariate approach to incorporate additional data sets). By comparing models, we were able to determine how choices made in building our integrated model affected the model's predictive performance. Our reference model is a joint likelihood model of local occurrence (Fletcher et al. 2019; Miller et al. 2019). The occupancy state *z* at site *i* can be described as:

(1) $$z_{i}\sim \text{Bernoulli}\left(\psi_{i}\right),$$

where $\psi_{i}$ is the probability that a species is truly present at site *i*. This probability is modeled as a linear function of covariates such that:

(2) $$\text{logit}\left(\psi_{i}\right)=\mathrm{\beta X}$$

where β is a vector of coefficients for the intercept and covariates ($X=\left[1,X_{1},\ldots ,X_{n}\right]$).

**TABLE 1:** We developed a candidate set of distribution models that allowed us to compare approaches to account for variable effort and integration of less reliable data types using different data integration approaches.

Variable effort			
Name	Effort filter	Spatial balancing	Effort model included
R1	Standard	Yes	Yes
F1	None	Yes	Yes
F2	Standard	No	Yes
F3	None	No	Yes
F4	Standard + removed traveling checklists	Yes	Yes
F5	Standard	Yes	No
F6	None	No	No

Data reliability		
Name	Less reliable data set	Integration method
R1	—	Joint likelihood
E1	eBird	Joint likelihood + false positive rate
E2	eBird	Covariate
E3	eBird	eBird removed
R2	—	Joint likelihood
O1	Historic data	Joint likelihood + false positive rate
O2	Historic data	Covariate
O3	Historic data	Old data removed

We estimated this relationship using a generalized additive model (GAM) to accommodate non‐linearity in covariate relationships. The number of basis functions *k* comprising the predicted curve was set to 10 for all covariates except the spatial interaction term which was set to 100. To limit over‐fitting, parameters were penalized for overfitting using a Bayesian parameter *λ* with a gamma prior, analogous to penalties used in REML fitting using likelihood approaches (Pedersen et al. 2019). The priors for each smoothed parameter were generated using the jagam function in the mgcv package (version 1.8–33; Wood 2017) in R (R Core Team 2021).

#### 2.2.2 Observation Model: BBS (Y)

The observed data for site *i* is denoted $Y_{i}$, where $Y_{i}=1$ if the species was detected and 0 if the species was undetected. The probability for *Y* is denoted as:

(3) $$Y_{i}\sim \text{Bernoulli}\left(z_{i}*p_{i}^{\mathrm{BBS}}\right),$$

where $z_{i}$ is the occupancy state of site *i* and $p_{i}^{\mathrm{BBS}}$ is the probability the species is observed given $z_{i}$ = 1 (i.e., the detection probability). We account for variation in the detection probability for BBS data based on the day of the year that the survey was conducted, which can affect detection of bird species (Skirvin 1981; Best 1981). The detection probability for BBS data is estimated as:

(4) $$\text{logit}\left(p_{i}^{\mathrm{BBS}}\right)=\;\alpha_{0}+\alpha_{1}*\mathrm{day}+\alpha_{2}*{\mathrm{day}}^{2},$$

where $\alpha_{0}$ is the intercept and $\alpha_{1}$ and $\alpha_{2}$ are the coefficients for the day of year effect.

#### 2.2.3 Observation Model: BBA (W)

The observed state of the BBA data ($W_{i}$) is a function of the number of intervals in which an individual was detected, ranging from 0 to 5. Because counts were recorded for five intervals, $W_{i}$ is binomially‐distributed with *n* = 5 and probability $z_{i}*p_{i}^{\mathrm{BBA}}$ such that,

(5) $$W_{i}\sim \text{Binomial}\left(z_{i}*p_{i}^{\mathrm{BBA}},5\right)$$

where $p_{i}^{\mathrm{BBA}}$ is the probability an individual is detected given the site is occupied ($z_{i}=1$). We again included day of year and time of day, which was represented as hours since midnight in our model for detection, so that:

(6) $$\text{logit}\left(p_{i}^{\mathrm{BBA}}\right)=\;\gamma_{0}+\gamma_{1}*\text{time}+\gamma_{2}*{\text{time}}^{2}+\gamma_{3}*\mathrm{day}+\gamma_{4}*{\mathrm{day}}^{2}.$$

#### 2.2.4 Observation Model: eBird (V)

eBird data can be modeled using the same structure as BBS data, where the observed occupancy state is Bernoulli‐distributed with probability $z_{i}*p_{i}^{\text{eBird}}$.

(7) $$V_{i}\sim \text{Bernoulli}\left(z_{i}*p_{i}^{\text{eBird}}\right),$$

To account for imperfect detection, we included an estimate of effort in the model for eBird data. In this case, $p_{i}^{\text{eBird}}$, or the conditional detection probability, is modified such that

(8) $$p_{i}^{\text{eBird}}=1-{\left(1-p_{*}\right)}^{E}$$

(9) $$E=\delta_{1}*\text{duration}+\delta_{2}*\text{distance},$$

where *E* is survey effort and the effort coefficients (δ) are constrained to be greater than 0. Stauffer et al. (2018), Miller et al. (2019), and Zulian et al. (2021) describe this parameterization for survey effort in detail. Longer survey duration or greater distance traveled indicates greater effort, which in turn increases the detection rate. The function assumes a constant rate of detection per unit of survey effort and that detection will approach 1 as E increases and 0 as duration and distance approach 0. We standardize effort using the detection probability of $p_{*}$ = 0.5, so that when E is estimated to be 1, the probability of detecting the species in any given interval is 0.5.

### 2.3 Model Set 1: Accounting for Effort With eBird Data

We evaluated two general strategies for addressing heterogeneous effort in eBird data (variable‐effort models; Table 1). The first strategy involved filtering to standardize effort or enforce spatial balance. Our baseline model (R1) applied the standard eBird effort filters and spatial balancing (see Data section). We then tested four alternative filtering schemes: (F1) removing all effort filters, (F2) removing spatial balancing, (F3) removing both effort filters and spatial balancing, and (F4) adding a filter to exclude traveling checklists in addition to the standard filters and spatial balancing. In all cases, only the eBird dataset used for model fitting was modified; the model structure remained unchanged.

The second strategy altered the model structure by removing explicit accounting for individual survey effort (Equations 8 and 9), effectively assuming equal detection probability across all checklists regardless of duration or distance. We fit this parameterization using eBird data with standard filters and spatial balancing (F5; same data as R1) and without any effort filters or spatial balancing (F6; same data as F3).

### 2.4 Model Set 2: Integrating Less Reliable Data

Next, we compared a set of models chosen to assess the best approach to integrate data sets when one is considered less reliable data than the other data sets. These models (data reliability models, Table 1) were again compared to a reference model. We considered three alternative approaches: a joint likelihood model that allowed for false‐positive detections in the observation model, a covariate model in which the less reliable data were incorporated as a covariate, or completely removing the less reliable data and estimating distributions solely with the reliable data sources.

We used two data sets to examine strategies for integrating less reliable data. The first was for eBird, which may be less reliable for fitting models than the BBS and BBA, due to its semi‐structured sample design. Models using eBird as the less reliable data set were compared to the reference model described above (R1). We also considered cases where data collected prior to our period of interest for inference (current data: 2015–2018) may be less reliable for predicting the current range (Table 1). We define “historic data” as data from all sources prior to 2015 and “current data” as data from 2015 to 2018, which only includes data collected by eBird and the BBS (i.e., all BBA data [2004–2009], and some eBird [2004–2014] and BBS data [1997–2014], were collected prior to 2015). The models that treated historic data as the less reliable data set were compared to a second reference model (R2). R2 maintained the same model structure and treatment of eBird data as R1 (i.e., effort filtering, spatial balancing, effort model) but was trained and validated using different temporal subsets of the data (see *Model testing and validation*). We test the same three integration strategies for each of these data sets.

Our first approach was to account for reliability as a source of observation error by incorporating a false positive detection rate into the respective observation model (E1, O1; Miller et al. 2013, Pacifici et al. 2017). To do this, we modified the probability a site is not occupied ($1-z_{i}$) by multiplying the probability by a false positive rate, f*, such that,

(10) $$Y_{i}\sim \text{Bernoulli}\left(z_{i}*p_{i}+\left(1-z_{i}\right)*f_{i}^{*}\right)$$

(11) $$f^{*}=1-{\left(1-f\right)}^{E_{i}}$$

where the false positive rate is estimated using a uniform prior constrained from 0 to 0.25 and *E* remains the measure of survey effort (i.e., the probability of generating a false positive error increases with greater survey effort). The prior was constrained to avoid identifiability issues, and posterior checks ensured that the boundary values were not included (Miller et al. 2011).

For our second approach, we treated the less reliable data set (eBird and historic data) as a covariate (E2, O2) and jointly modeled the reliable data sources (BBS and BBA in E2; current BBS and current eBird in O2; Pacifici et al. 2017). The covariate model treats observations as a covariate that can then be used as a predictor variable in the species distribution model fit using the other data sets. If the covariate is a poor predictor of the other data sets, it is given little weight in the distribution model, whereas if it is a strong predictor, it will be given a greater weight in predictions. To produce a covariate from the eBird data, a 1000‐m buffer was created around each BBA and BBS point, and the number of eBird checklists and count of the given species were summarized within the buffer. Thus, the updated expression for the probability of occupancy at site *i* is:

(12) $$\begin{array}{c} \text{logit}\left(\psi_{i}\right)=\beta_{0}+\beta_{1}*X_{1}+\beta_{2}*X_{2}+\ldots \beta_{n}*X_{n}\\ \;+\beta_{\text{count}}*\text{count}+\beta_{\text{lists}}*\text{lists} \end{array}$$

We constrained $\beta_{\text{count}}$ and $\beta_{\text{lists}}$ to be positive and negative, respectively, to ensure a positive predictor for occupancy that is discounted by the amount of effort or number of lists (Pacifici et al. 2017; Miller et al. 2019). Similarly, we summarized the historic data as the total species count and the number of lists within a 1000‐m buffer around each eBird and BBS point to produce a covariate from the historic data.

The final approach we considered was simply to remove the less reliable data set entirely (I3, O3) to determine whether model performance is best with only reliable data.

### 2.5 Model Testing and Validation

We fit all models using Markov chain Monte Carlo analysis implemented using JAGS (v. 4.3.0; Plummer 2003), called from the jagsUI package in R (v1.5.1; Kellner 2019). Three chains were run for 2500 iterations with a burn‐in of 500 iterations, and a thinning of 2, producing a total of 1000 posterior samples per chain.

We assessed the predictive performance of models using cross‐validation (Hooten and Hobbs 2015), dividing our data into training and testing data sets. For models F1‐F6, E1‐E3, and for R1, 20% of the BBA and BBS data sets were excluded from model fitting and treated as testing data for validation. We chose to validate models using BBA and BBS data because these were our structured data sets and thus better suited for assessing performance of the distribution model. For models O1‐O3 and the R2 reference model, we selected 20% of the current data (BBS and eBird from 2015 to 2018) for the testing data. The remaining current data and all historic data included in the scenario were used to train the model.

We calculated two measures of predictive performance: Brier score and area under the receiver‐operating‐curve (AUC), measures of calibration and discrimination, respectively (Norberg et al. 2019). Brier score is equivalent to the mean squared error between the predicted values and the binary outcome (Brier 1950) and was calculated using observed and predicted observations. Brier scores measure absolute differences between predictions and actual values for test data. AUC was calculated by determining the proportion of all sample combinations where the predicted probability the species occurred was greater in a sample where the species was observed than in a sample where it was not observed. AUC measures how well the model discriminates between binary presence‐absence outcomes (Hanley and McNeil 1982; Broms et al. 2016). We calculated this value using the package ROCR (Sing et al. 2005).

## 3 Results

The amount of data available to fit models varied among scenarios (Figure S1). There was also a wide range in the frequency with which individual were detected among the four species tested (Figure S2). Wood Thrush was the most common (18%–26% of observations included positive detections across the three data types). Golden‐winged Warbler (0.13%–0.47%), Canada Warbler (0.37%–1.7%), and Cerulean Warbler (0.51%–1.2%) were detected at much lower rates. Across all species, the proportion of observations that included a species was highest in eBird, although when traveling checklists were excluded (F4), eBird detection rates were closer to BBS and BBA rates.

**FIGURE 1:**
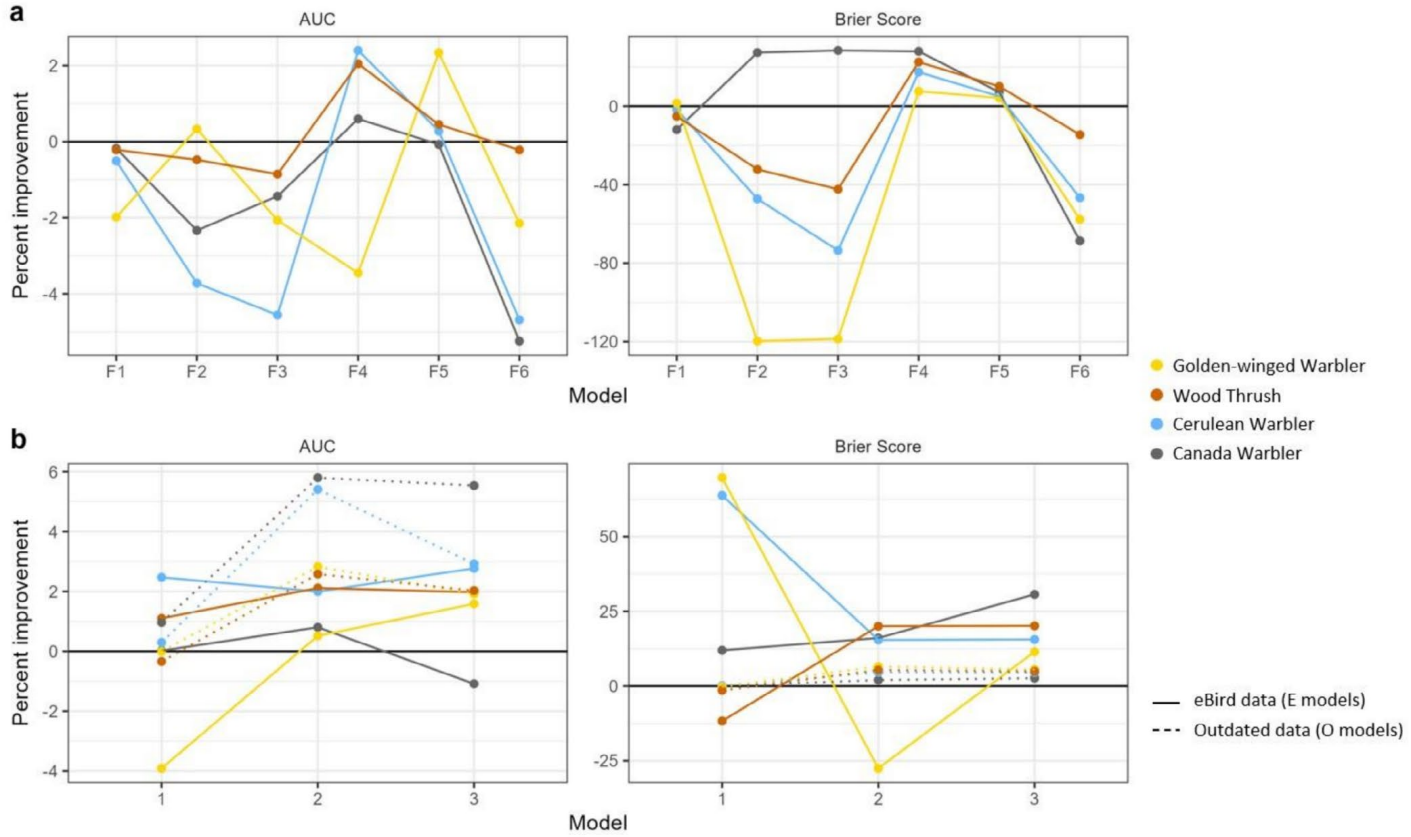
Percent improvement in AUC and Brier Score for each model relative to the reference model, which is represented with the horizontal black line. Methods for accounting for variable effort in eBird data are shown in (a); methods for integrating less reliable data are shown in (b). In b, models in which eBird data is the less reliable data set are represented with a solid line and are compared to reference model R1, whereas models in which historic data is the less reliable data are represented with a dotted line and are compared to reference model R2. Percent change was calculated as the difference between the validation metric values of the reference model and each alternative model, divided by the value of the reference model. Percent change of Brier Score were multiplied by −1 so that positive values indicate improvement for both metrics.

When considering how to best account for eBird effort, we found relatively consistent results across the four species (Figure 1a, Table 2). The most common best performing model, measured by both Brier scores and AUC was F4, which excluded traveling eBird checklists (i.e., distance = 0 for all checklists) in addition to the standard effort filters and spatial balancing. Models that excluded spatial balancing (F2, F3, F6) tended to perform far worse than the reference model. Canada Warbler was an exception to this pattern: models F2 and F3 performed better than the reference model according to Brier Score. For 3 of the 4 species, accounting for effort in estimating detection probability had little or no predictive benefit, with the one exception being the model for Golden‐winged Warbler, whose performance benefitted from removing the effort model (F5).

**TABLE 2:** Validation metrics for methods of accounting for variable effort in eBird data.

Model	Canada Warbler		Cerulean Warbler		Golden‐winged Warbler		Wood Thrush	
	Brier Score	AUC	Brier Score	AUC	Brier Score	AUC	Brier Score	AUC
R1	0.0046	0.871	0.0073	0.8682	0.0020	0.9065	0.3277	0.7083
F1	0.0052	0.8695	0.0075	0.8638	0.0002	0.8885	0.3446	0.7067
F2	0.0034	0.8507	0.0108	0.8359	0.0044	0.9095	0.4332	0.7049
F3	**0.0033**	0.8585	0.0127	0.8286	0.0044	0.8878	0.4664	0.7022
F4	**0.0033**	**0.8762**	**0.0061**	**0.889**	**0.0019**	0.8752	**0.2539**	**0.7228**
F5	0.0043	0.8704	0.0070	0.8706	**0.0019**	**0.9277**	0.2947	0.7114
F6	0.0078	0.8253	0.0107	0.8275	0.0032	0.8870	0.3753	0.7067

*Note:* Model(s) with best fit are highlighted in bold.

When integrating less reliable data, we found different patterns depending on which data set was treated as less reliable (Figure 1b, Table 3). In both cases, using the reliable data set as a covariate (E2, O2) and eliminating it entirely (E3, O3) generally improved predictive performance (AUC and Brier Score) compared to the reference model. Adding a false positive rate for eBird data (E1) generally improved performance relative to the reference model but doing the same for historic data (O1) made little difference.

**TABLE 3:** Validation metrics for methods to account for incorporation of less reliable data.

Model	Canada Warbler		Cerulean Warbler		Golden‐winged Warbler		Wood Thrush	
	Brier Score	AUC	Brier Score	AUC	Brier Score	AUC	Brier Score	AUC
R1	0.0046	0.8710	0.0073	0.8682	0.0020	0.9065	0.3277	0.7083
E1	0.0041	0.8712	**0.0027**	0.8897	**0.0006**	0.8710	0.3660	0.7160
E2	0.0039	**0.8780**	0.0062	0.8855	0.0026	0.9112	0.2620	**0.7232**
E3	**0.0032**	0.8615	0.0062	**0.8923**	0.0018	**0.9209**	**0.2617**	0.7222
R2	0.0138	0.8396	0.0121	0.8938	0.0042	0.9452	0.1432	0.7872
O1	0.0138	0.8477	0.0121	0.8965	0.0042	0.9452	0.1453	0.7845
O2	**0.0135**	**0.8883**	**0.0116**	**0.9422**	**0.0039**	**0.9720**	**0.1355**	**0.8075**
O3	**0.0135**	0.8861	**0.0116**	0.9200	0.0040	0.9634	0.1362	0.8031

*Note:* Model(s) with best fit are highlighted in bold.

**FIGURE 2:**
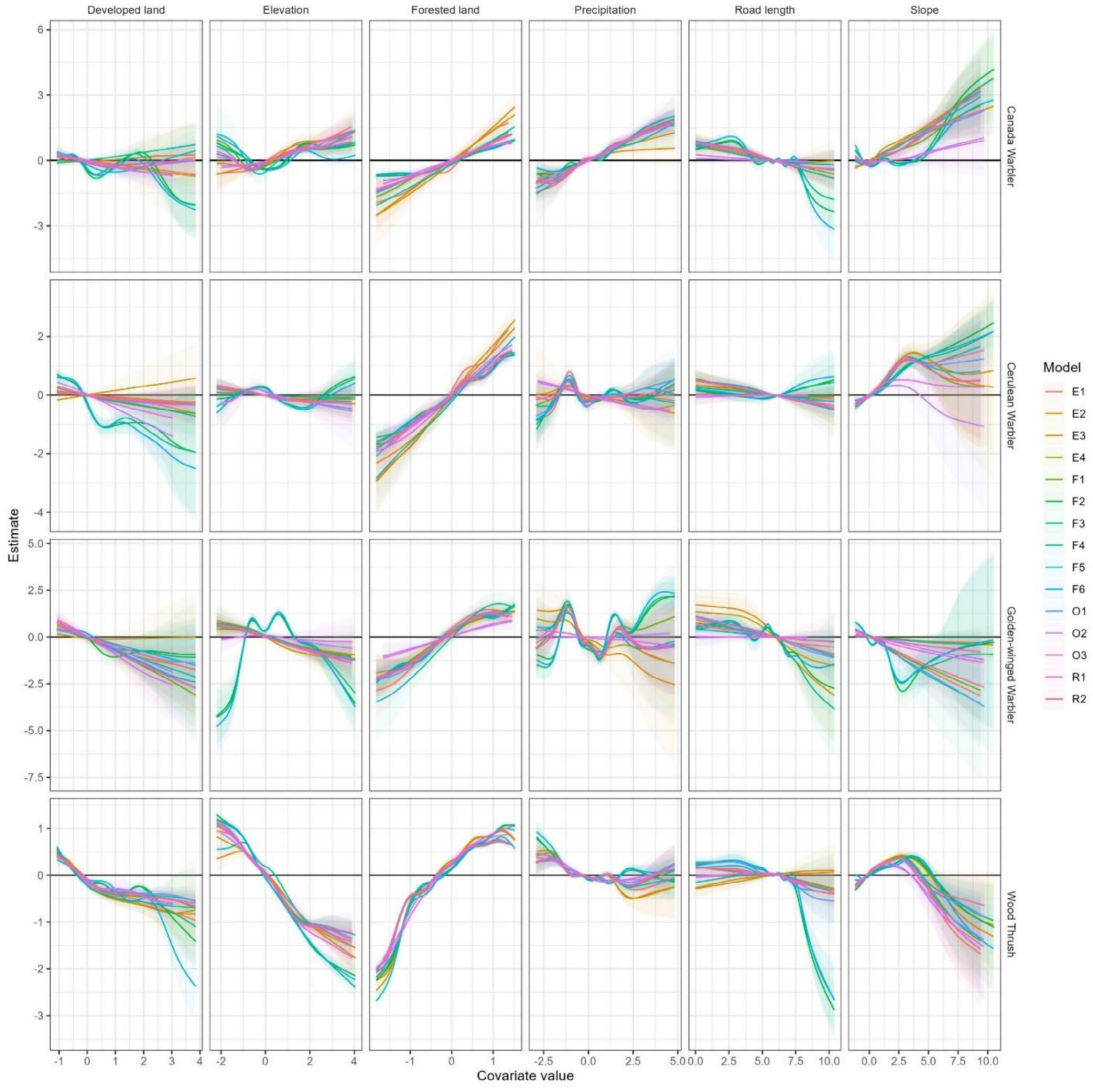
Covariate relationships for each species from each model. The solid black line indicates when the coefficient estimate is zero, and there is no effect. Solid colored lines represent the coefficient value by species. Shaded area represents the 95% credible interval.

**FIGURE 3:**
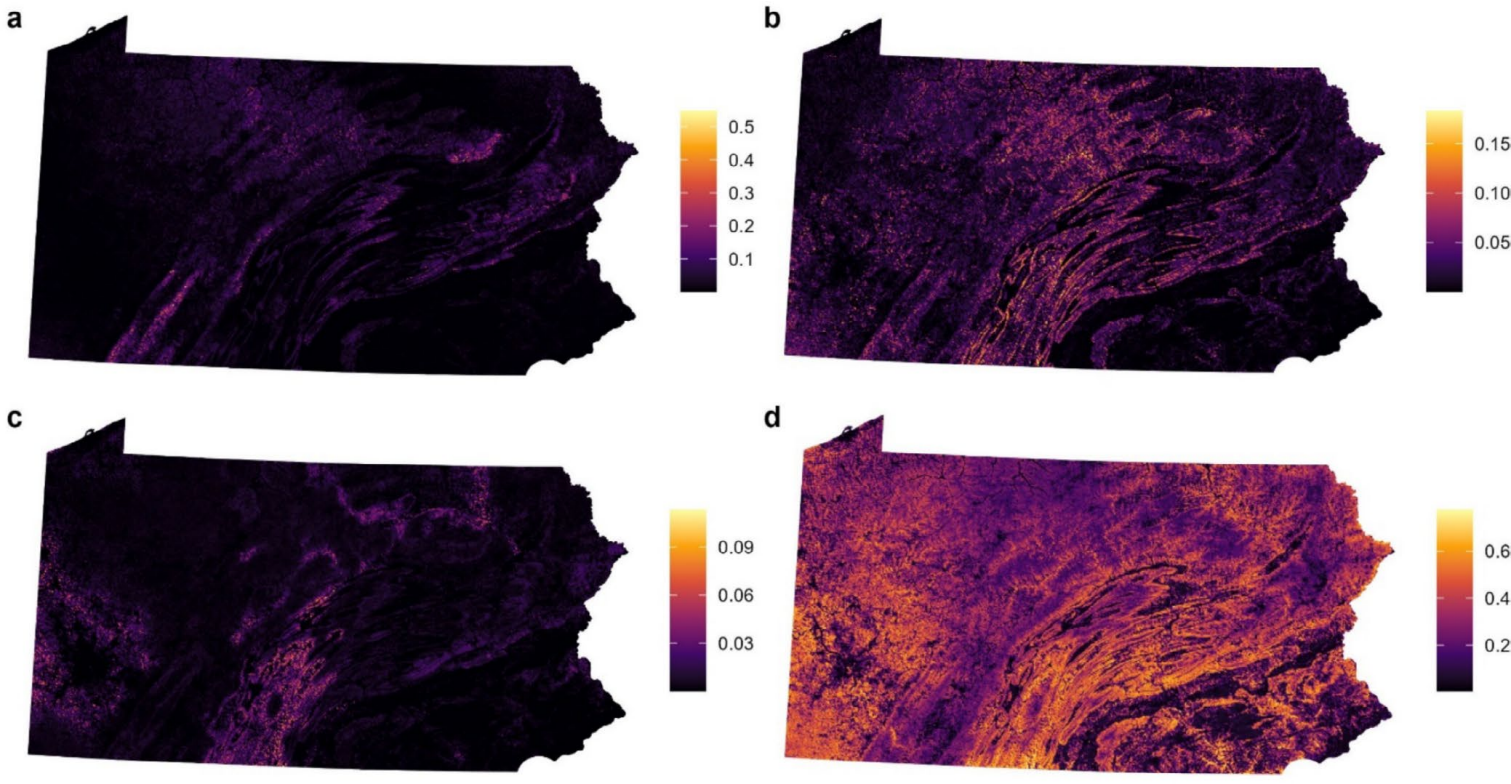
Posterior predicted occupancy probabilities for (a) Canada Warbler, (b) Cerulean Warbler, (c) Golden‐winged Warbler, and (d) Wood Thrush based on reference model R1. Occupancy is estimated within 1 × 1‐km grid cells within Pennsylvania.

Covariate relationships were consistent across most models (Figure 2). In some cases, models F2, F3, and F6, which excluded spatial balancing, identified covariate relationships that were consistent with each other but different from other models. All species had a positive relationship with forested land and generally negative relationships with developed land and total road length, with Golden‐winged Warbler being most negatively associated with these measures of anthropogenic habitat modification. Relationships with elevation and slope differed among species: both covariates had negative relationships with Golden‐winged Warbler and Wood Thrush, where these species were more abundant in flat low‐elevation sites. In contrast, both were positively associated with Canada Warbler occurrence. Covariate relationships from model R1 were used to produce the species distribution maps in Figure 3.

## 4 Discussion

Our modeling efffort and analyses can be used to inform others implementing integrated species distribution models. First, we demonstrate that predictive accuracy can improve when methods explicitly account for uncertainty. Specific to the use of eBird data, spatial balancing as recommended by Strimas‐Mackey et al. (2020) was particularly important in dealing with variation in effort, and removal of traveling eBird checklists can further improve predictions. Our results suggest that strict rules for filtering that control for survey specific and spatial effort are an important strategy to improve prediction when sample effort is highly clustered.

Accounting for data reliability also improved results, though patterns differed for the two case studies (i.e., participatory data versus historic data). For historic data, treating the less reliable data as a covariate performed best and led to the greatest improvement in predictive accuracy, while having the added benefit of being more computationally efficient. For eBird data, results varied greatly across species, but models that addressed reliability performed better than reference models that did not.

We first examined methods to account for variable effort in eBird data. We found that our reference model, which used effort filters and spatial balancing as recommended by Strimas‐Mackey et al. (2020) and contained an effort model for eBird, generally performed better than or as well as alternative methods to account for eBird effort. Our results indicate that spatial balancing is likely the most important component driving model performance. The model that removed effort filters but maintained spatial balancing performed similarly to the reference model, whereas all models that removed spatial balancing, regardless of whether effort filters were used or not, performed much worse. Models that removed spatial balancing also identified covariate relationships that were not always consistent with other models. The inclusion of an effort model had minimal effect on model performance when spatial balancing and effort filters were maintained. When the filters and balancing were removed in addition to the effort model, however, performance declined. Interestingly, we found that predictions can be improved by removing traveling eBird checklists in addition to the standard filtering and balancing. This benefit may come from reducing the spatial uncertainty in locations that comes with non‐stationary data collection.

We identified somewhat different patterns depending on which data set was treated as the less reliable data set. Using a false positive rate for eBird data improved model performance for all species except Wood Thrush, whereas using a false positive rate for historic data had little effect on model performance. Our results provide consistent support for the benefits of using less reliable data as a covariate in the model, which has an important advantage of also being less computationally intensive (Pacifici et al. 2017; Miller et al. 2019; Fletcher et al. 2019). Predictive accuracy is improved when either historic data or eBird data are used as covariates.

Our analysis helps understand the benefit from older data in models that predict current species distributions, which can be uncertain especially if the species has experienced range shifts. For the four species in our analysis, our results suggest that historic data is best used as a covariate or not at all. However, species population trends relative to the age of the data set could be a consideration when determining the utility of historic data for static models. For example, model performance for Golden‐winged Warbler improves when old data are excluded. This is likely because this species has experienced significant habitat changes and is thus likely occurring in a different environment now than a decade ago (Swarthout et al. 2009; Rosenberg et al. 2016). Older data such as museum specimens may be useful, however, for a species whose range has been somewhat consistent over the timescale of the data.

Integrating multiple data streams has the potential to greatly increase the accuracy of species distribution models, which will improve understanding of species ecology and conservation status. Data integration methods allow the use of less reliable data sources, such as large participatory science‐based data sets and historical records. It also provides a framework for properly accounting for patterns of sample effort, which effect observations from individual data sets. We demonstrate that combining semi‐structured eBird and older BBA data sets, yields the best predictive performance appropriate filtering and integrate steps are applied. These findings highlight the value of incorporating less reliable sources within integrated species distribution models and provide guidance for best practices for data integration that extend beyond the species and data sets used here.

Our findings contribute to a growing literature on integrated species distribution models, which aim to combine diverse data sources to improve ecological inference and conservation planning. Similar challenges of data reliability and sampling bias occur across taxa. For example, studies on butterflies (Pagel et al. 2014), amphibians (Zipkin et al. 2017), birds (Stauffer et al. 2018; Zulian et al. 2021), and trees (Fithian et al. 2015) have shown that accounting for effort and data quality improves predictions. Work on plants and insects (Rakosy et al. 2022; Davis et al. 2023) has also highlighted the benefits of integrating historical records with contemporary citizen‐science data. The strategies we identify, such as treating less reliable data as covariates and applying strict filtering, are generalizable, though additional work will be needed to determine how species ecology, detectability, and data characteristics affect results. Future research should evaluate these approaches across taxa to continue to establish best practices for data integration.

## Author Contributions

**Fiona Lunt:** writing – original draft (equal). **C. Lane Scher:** writing – original draft (equal). **Riley O. Mummah:** writing – original draft (equal). **David A. W. Miller:** writing – original draft (equal).

## Disclosure

Any use of trade, firm, or product names is for descriptive purposes only and does not imply endorsement by the US government.

## Conflicts of Interest

The authors declare no conflicts of interest.

## Supporting information


**Data S1:** Supporting Information.

## Data Availability

All data used in this analysis are publicly available. Species data were downloaded from publicly available databases maintained and served by eBird (eBird 2019), the Breeding Bird Survey (Pardieck et al. 2020), and the Breeding Bird Atlas (Paton et al. 2019). Environmental data were taken from a variety of publicly available sources that are detailed in the manuscript. All code and data needed to conduct the analysis is available on GitHub (https://github.com/lanescher/BirdSDM‐D529/README.md).
