# Supplementary material for: Incorporating Data Sets With Multiple Sources of Uncertainty in Integrated Species Distribution Models
Source: Ecol Evol. 2026 Apr 9;16(4):e73185. doi: 10.1002/ece3.73185 (PMC13065488; doi:10.1002/ece3.73185)

**Supplemental Figures**

Figure S1. Number of observations from each source used to fit models. Observations that were used to generate covariates (E2 and O2) are not shown.


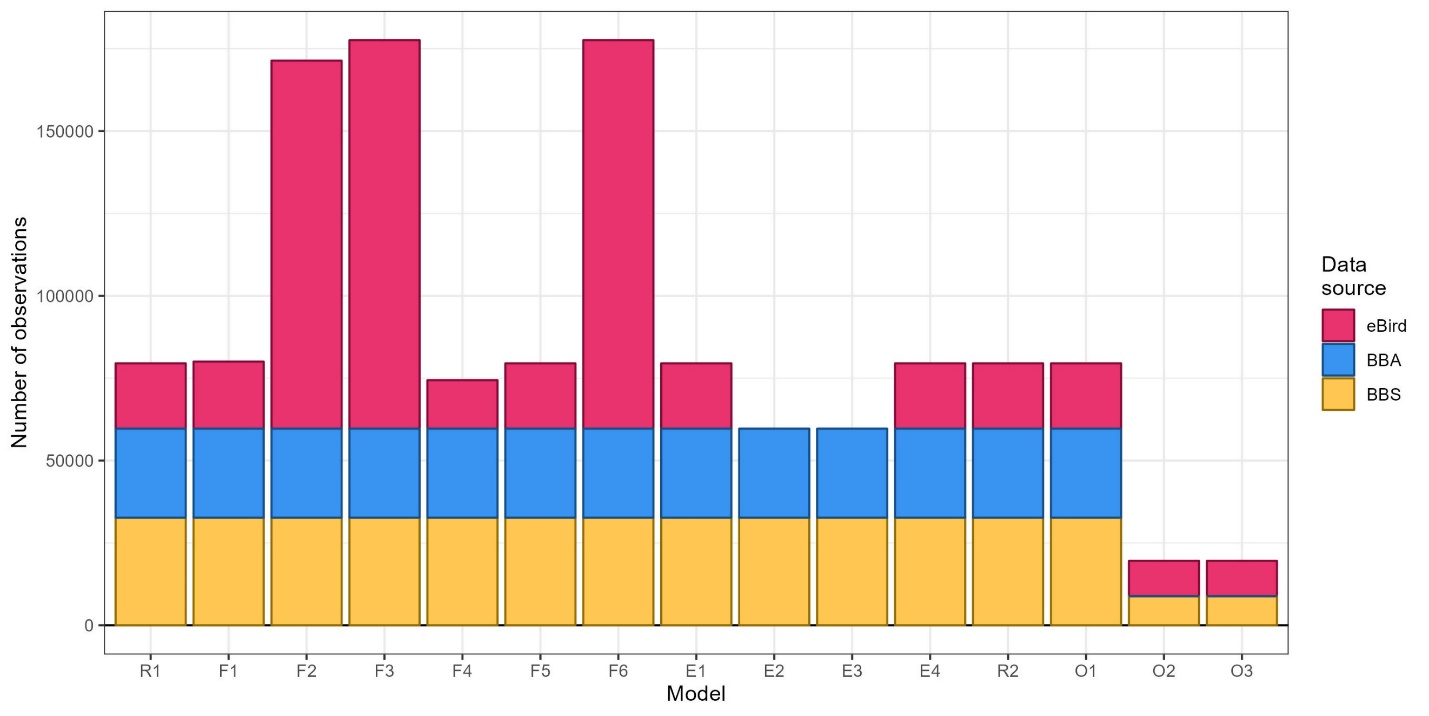


Figure S2. Detection rate of each species in each data set.


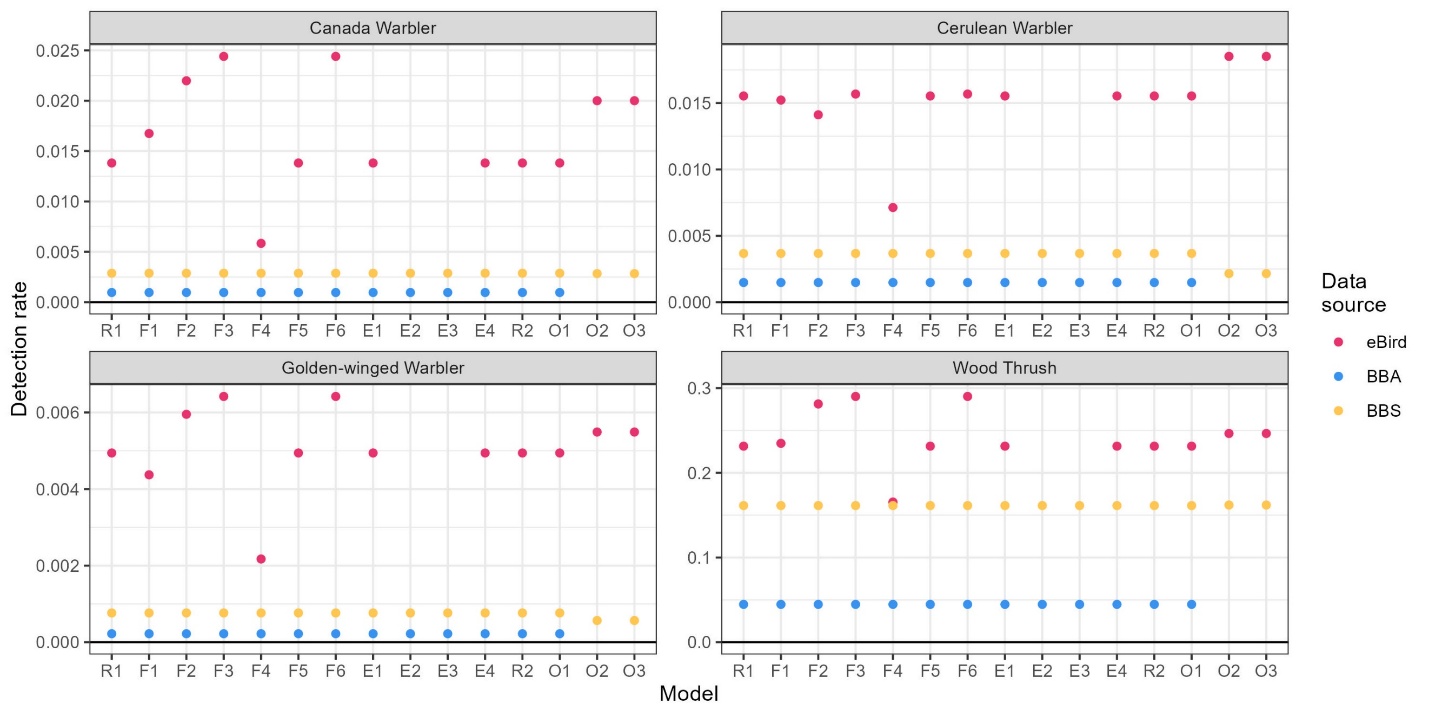

Supplement: Supplementary file 1 — Data S1: Supporting Information. [file ECE3-16-e73185-s001.docx]
